# Protein Elicitor PeBL1 of *Brevibacillus laterosporus* Enhances Resistance Against *Myzus persicae* in Tomato

**DOI:** 10.3390/pathogens9010057

**Published:** 2020-01-14

**Authors:** Khadija Javed, Dewen Qiu

**Affiliations:** The State Key Laboratory for Bio-Pesticides Engineering of Plant Disease Biocontrol and Insect Pests, Institute of Plant Protection, Chinese Academy of Agricultural Science, No. 12 Zhong-Guan-Cun South Street, Beijing 100081, China; khadijajaved829@gmail.com

**Keywords:** PeBL1, aphid resistance, salicylic acid pathway, jasmonic acid pathway, defense response, feeding behavior

## Abstract

*Myzus persicae*, a destructive aphid of tomato usually managed by chemical pesticides, is responsible for huge annual losses in agriculture. In the current work, a protein elicitor, PeBL1, was investigated for its capacity to induce a defense response against *M. persicae* in tomato. Population growth rates of *M. persicae* (second and third generation) decreased with PeBL1 treatments as compared with controls. In a host selection assay, *M. persicae* showed preference for colonizing control plants as compared to tomato seedlings treated with PeBL1. Tomato leaves treated with PeBL1 gave rise to a hazardous surface environment for *M. persicae* due to formation of trichomes and wax. Jasmonic acid (JA), salicylic acid (SA), and ethylene (ET) showed significant accumulation in tomato seedlings treated by PeBL1. The following results showed that PeBL1 significantly modified the tomato leaf surface structure to reduce reproduction and deter colonization by *M. persicae*. Defense processes also included activation of JA, SA, and ET pathways. The study provides evidence for use of PeBL1 in the protection of tomato from *M. persicae*.

## 1. Introduction

During the course of evolution, a complex relationship has formed between plants and herbivores. Plants damaged by herbivores show accumulation of toxic or volatile organic compounds with modification of their physical structures. These compounds and structures affect colonization by herbivores and their development, feeding, survival, and oviposition, which in turn attract natural enemies and induce defense [1]. Two defense mechanisms, mainly constitutive, have been developed by plants to deal effectively with this damage [2]. Physical barriers, including cuticle trichomes, callose, cell walls, and suberin, prevent plants from being colonized, whereas allelochemicals with antibiotic effects affect pest development, growth, fecundity, and durability of insects, or induce repellent effects [3].

Aphids are phloem-feeding insects, which transmit plant viruses by consuming plant sap, thus resulting in severe agricultural losses [4,5]. Defense responses induced by aphids have been analyzed in various aphid–plant systems. Green peach aphid showed decreasing fecundity in infested leaves of *Arabidopsis thaliana* [6]. In chilli plants, feeding caused confronting effects and increased emission of volatile organic compound, with a repulsive outcome versus infesting *Bemisia tabaci* [7]. Reduction in survival rate and population growth parameters of immature *Plutella xylostella* were found in *Brassica napus* due to resistance to *Brevicoryne brassicae* [8].

Jasmonic acid (JA), salicylic acid (SA), and ethylene (ET) are involved in the induction of a defense response in plants [9]. SA was found to be involved in defense against piercing–sucking insects, whereas JA against chewing insects [10]. ET controls various processes that are associated in defense responses in plants [11,12]. Monarch caterpillars, *Danaus plexippus*, increased activation of the JA pathway, but balanced acquisition in SA whereas the oleander aphids, *Aphis nerii*, caused an opposite effect in *Asclepias syriaca* [12]. Few previous studies have shown involvement of JA and SA in the induction of an aphid-response in plants due to enhanced expression of genes encoding PR-1 (a SA pathway marker protein: pathogenesis related protein 1), ICS (isochrorismate synthase), AOS (allene oxide synthase), and FAD (Ω-3 fatty acid desaturase), that were noticed to be related to SA- and JA-induced responses, after aphid feeding [13,14].

The green peach aphid (*M. persicae*), a severe pest of tomato, cucumber, maize, barley, wheat, and beans in China, has a direct effect on the yield and quality of crops because of its feeding behavior. Defense response in plants is triggered by biotic and abiotic elicitors [15]. The Elicitors are associated with several pathogens such as fungi, bacteria, viruses, and oomycetes. The elicitors are mainly proteins, glycoproteins, peptides, lipids, and oligosaccharides [16]. They mainly form two major groups including race-specific ones that trigger a defense response in host plants only, and those inducing a general defense response in both host and non-host plants [17]. Abiotic elicitors are inorganic chemicals and metals, with other abiotic stress factors. Biotic elicitors are produced by the attack of pathogens or herbivores [18]. Due to an increase in the demand of food safety and quality, elicitors have been studied as a replacement for some chemical pesticides [19].

PeBL1 is a broad-spectrum elicitor studied in *Brevibacillus laterosporus* strain A60 that has been found to activate resistance in plants through the JA, SA, and ET pathways. Its activity results in the activation of defense enzymes, strengthening the cell wall, and up regulation of other defense related genes [20]. Pathogenicity of *B. laterosporus* has been linked to a combination of sporulated cultures with/without parasporal bodies, and is active against dipterans such as flies and mosquitoes [21,22]. *Brevibacillus laterosporus*, an antimicrobial species and invertebrate pathogen, is characterized morphologically by a typical spore, surrounded by a tightly attached canoe-shaped parasporal body. Biocontrol potential in *B. laterosporus* is not only restricted to insects, nematodes, and mollusks but also comprises phytopathogenic fungi and bacteria [23,24]. Biocontrol potential in entomopathogenic species has been analyzed on insects in the orders Coleoptera, Lepidoptera, and Diptera [21,24]. In the current study, function and mechanism of PeBL1 effects on tomato aphid control was studied by applications of the elicitor to tomato seedlings. Observations of trichomes on the leaf surface structure, contents of JA, SA, and ET and gene expression of JA, SA and ET were carried out to evaluate PeBL1’s potential effect on *M. persicae*. Data on function and mechanism of PeBL1 effects in tomato aphid control are herein provided.

## 2. Materials and Methods

### 2.1. Evaluation of PeBL1 against Aphid in Tomato Plant

Tomato aphid (*M. persicae)* was reared on tomato (*Solanum lycopersicum*) plants in a chamber at 60% relative humidity (RH), 23 ± 1 °C and, a photoperiod of 16:8 h light/dark, over 4 months. Tomato seeds were surface sterilized using 75% ethanol for 30–40 s and then washed with distilled water, and pre-soaked in water for 2–3 days before use. PeBL1 was produced in *Escherichia coli* BL21-DE3 using the recombinant vector pET30-TEV/LIC (Novagen, Darmstadt, Germany). Purification of PeBL1 was performed with His-Trap HP column (GE Healthcare, Waukesha, WI, USA), and in a HiTrap desalting column (GE Healthcare, Waukesha, WI, USA), as described by Wang et al. [20].

### 2.2. *Myzus persicae* Population

Young seedlings were soaked for 24 h in four different concentrations of PeBL1 solution, i.e., 88.72, 53.23, 26.61, and 22.17 μg mL^−1^. Three seeds in a single pot were grown in organic soil (Flora guard SUBSTRAT). Three weeks-old tomato seedlings were sprayed with the concentrations of PeBL1 solution after 7 days, followed after 24 h by inoculation with 8–10 adults of *M. persicae* per plant. The number of aphids after inoculation was then recorded every 5 days. Positive and negative controls were tested with water and 88.72 μg mL^−1^ buffer (50 mM Tris-HCl, pH 8.0). Transparent air-permeable cages were used to separate seedlings of each pot from others. The experiment was performed twice with four replications.

### 2.3. Increase in Intrinsic Rate of *M. persicae* Population

Tomato seeds were soaked for 24 h in PeBL1 purified protein solution of 88.72 μg mL^−1^ and then were moved to petri dishes for germination for 2–3 days in distilled water. Seedlings were sprayed with PeBL1 purified protein solution of 88.72 μg mL^−1^ after 24 h. Inoculation with a newly born nymph of *M. persicae* was then performed for each seedling. A cotton-gauze-covered glass tube was used to separate every seedling from each other. The newly born aphid was observed twice a day to record the total time and number of offspring it produced that were then taken out each day. An identical test on seeds and seedlings were performed after 5 days. The experiment was individually repeated two times using 30 replicates per treatment. The increase of intrinsic rate of each aphid was measured by the formula:

(1) $$r_{m}=0.738\times (\ln Md)/T_{d},$$

where Md represents the number of new born nymphs in a development time equal to $T_{d}$ which is the period from the aphid birth till its first reproduction.

### 2.4. Feeding Preference

Seeds and seedlings of tomato were tested as listed above. Three-weeks-old seedlings of tomato were treated with PeBL1 solution. The positive and negative controls were treated with water and buffer of 88.72 μg mL^−1^ (50 mM Tris-HCl, pH 8.0), respectively. Transparent and breathable cage (60 × 60 × 60 cm) with leaves cross touching and a white cardboard bridge (12 × 4 cm) connecting the basal part of stems were used to place treated PeBL1 and control seedlings. Thirty adults of wingless *M. persicae* were released in the middle of the bridge. The experiment was repeated 15 times counting the numbers of aphids on each seedling after 24 h.

### 2.5. Aphids Bioassay

PeBL1 elicitor purified protein with different concentrations (i.e., 88.72, 53.23, 26.61, and 22.17 μg mL^−1^) as treatment, positive control (water only), and negative controls (Buffer 88.72 μg mL^−^^1^) were bio-assayed against *M. persicae* on tomato plants. Bradford assay was used to determine different protein concentrations. At three-leaf stage of tomato plant, around 2–3 mL of PeBL1 solution was applied with a precise spray bottle until the solution drained off from plants. Water and buffer (50 mM Tris-HCl, pH 8.0) were applied to positive and negative controls. Plants were allowed to dry overnight, and, on the next day, 3 to 5 numbers of freshly moulted (0–6 h), old adult winged aphids per leaf were allowed to feed on these plants. Nymphal development time was recorded, by consecutive observations at 3 h intervals until the completion of the bioassays for each instar, as the total number of offsprings produced by all aphid instars, whereas the longevity was considered as the number of days the aphids used to live on. Bioassays at three non-identical temperature regimes (16, 22, and 27 °C) were repeated three times individually, using ten replicates per each treatment.

### 2.6. Surface Structure Observation of Leaves

Seven-day-old seedlings and seeds of tomato plant were treated similarly as above. Seeds were soaked for 8 days, one day after the spraying of seedlings. The central part was obtained and tested for first leaves, and 3.5% glutaraldehyde diluted in 0.1 M phosphate buffer (pH 7.2) was used to store samples for about 48 h until use. All samples were washed clean in 0.1 M phosphate buffer (pH 7.2) five times, for about 15 min each time and then submerged in 1% osmic acid for about 2 h. An ethanol gradient of 100, 95, 90, 80, 70, 60, 50, and 30% was applied for 15 min. A Leica EM CPD030 (Critical point dryer, Leica Bio systems, Wetzlar, Germany) was used to perform critical point drying on all samples. A Hitachi model H-7650 transmission electron microscope was used for observation of all samples, with 10 replicates per treatment.

### 2.7. Determining Plant Hormone with HPLC/MS

Seven-day-old seedlings and seeds were treated as mentioned above. The aerial part of seedlings, weighing about 0.5 g, was used to extract JA, SA, and ET, as described earlier [25]. Around 20 μL of extract was inoculated into a high-performance liquid chromatography mass spectrometer (HPLC/MS; Shimazu Scientific Instruments, ODS-C^18^, 3 μm, 2.1 × 150 mm, Kyoto, Japan). HPLC analysis was carried out at a flow rate of 0.2 mL min^−1^, 60% methanol as a mobile state, with 4 °C sample temperature and 40 °C column temperature, a desolvation temperature of 250 °C set with a selected ion monitoring (SIM) in negative ion mode (SA, m/z: 137.00; JA, m/z: 209.05), 200 °C of heat block, 10 L min^−1^ drying gas flow rate, 1.5 L min^−1^ nebulizing gas flow, 1.30 kV voltage of detector, and interface voltage of −3.5 kV.

### 2.8. Q-RT-PCR

Kits from TransGen Biotech (Beijing, China) were used to extract RNA, synthesize cDNA, and conduct quantitative real-time polymerase chain reaction (Q-RT-PCR) (ABI 7500 Real-Time PCR System). Nano-photometer NP80 was used to check the RNA quality. Tested genes used for the SA pathway were SOLYC09g007900 (PAL5), SOLYC03g036480 (PAL1), SOLYC10g08618 (PAL2), SOLYC02g032850 (PAD4), SOLYC01g106620 (NPR1), SOLYC04g079890 (PR5), SOLYC07g064990 (SAMT), and SOLYC12g014500 (SAMT). For the JA pathway, the tested genes were SOLYC04g079730 (AOS), SOLYC02g085730 (AOC), SOLYC10g086220 (OPR), SOLYC12g094520 (4CL), SOLYC04g054890 (ACX), SOLYC12g007170 (AIM1), SOLYC09g091470 (KAT2), and SOLYC01g006560 (LOX12). For the ET pathway, the tested genes were SOLYC12g09900 (SAMS), SOLYC03g043890 (ACCS), SOLYC08g081555 (ACCS), SOLYC07g026650 (ACCO1), SOLYC10g009110 (SlERF3/LeERF3b), SOLYC08g079750 (ACC5), SOLYC06g071640 (TAR2), and SOLYC03g118780 (PR5). The 18S ribosomal gene was considered as internal reference [26]. The primers used are listed in Table A1 of Appendix A. The genes’ relative fold expression was evaluated by the use of 2-ΔΔCT method [27].

### 2.9. Data Analysis

Data collected for each treatment pair were statistically compared with the independent Levene’s test and two-tailed t-test. Data obtained from three or more treatments were statistically compared by least significant difference (LSD) and one-way analysis of variance (ANOVA). For statistical data analyses, Statistix version 8.1 (Analytical Software, Tallahassee, FL, USA) was used. Data on fecundity of aphids were square-root transformed prior to analysis. In order to take out differences, one-way factorial analysis of variance was performed among treatment factors such as the concentrations of PeBL1 elicitor and different temperature regimes, followed by least significant different test, at a probability of 95%. The expressions of genes (RT-qPCR) were obtained by the comparative CT (2^−∆∆CT^) method. Student’s *t*-test (α = 0.05) was used for comparing fold changes in the plant samples treated with elicitor and buffer.

## 3. Results

### 3.1. Evaluation of Recombinant PeBL1

**Figure 1:**
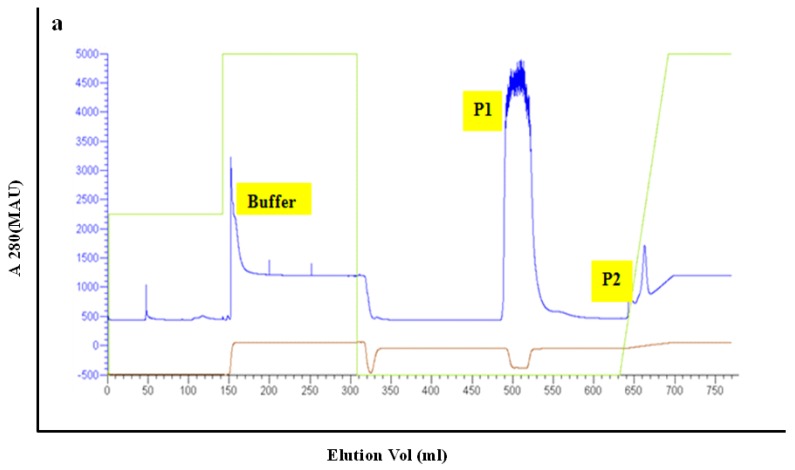
Purification of recombinant PeBL1. (**a**) Total *E. coli* proteins purification with a His-Trap HP column. Peak P2 includes recombinant PeBL1 eluted with the elution buffer (25 mM Tris, 200 mM NaCl, 500 mM imidazole, pH 8.0). Peak P2 was loaded on a HiTrap desalting column with 5 mL min^−1^ flow rate.

The PET30-TEV/LIC recombinant expression vector was transformed into *E. coli* BL21 (DE3) cells. After successful transformation, the expressed His6-PeBL1 was soluble in *E. coli*. PeBL1 was purified using a His-Trap HP column (GE Healthcare, Waukesha, WI, USA) (Figure 1a) and desalted in a HiTrap desalting column (GE Healthcare) as described by Wang et al. [20]. At 12 kDa on Tricine SDS-PAGE, a single band showed the characteristics of the pure recombinant protein.

### 3.2. Indoor Performance of *M. persicae*

**Figure 2:**
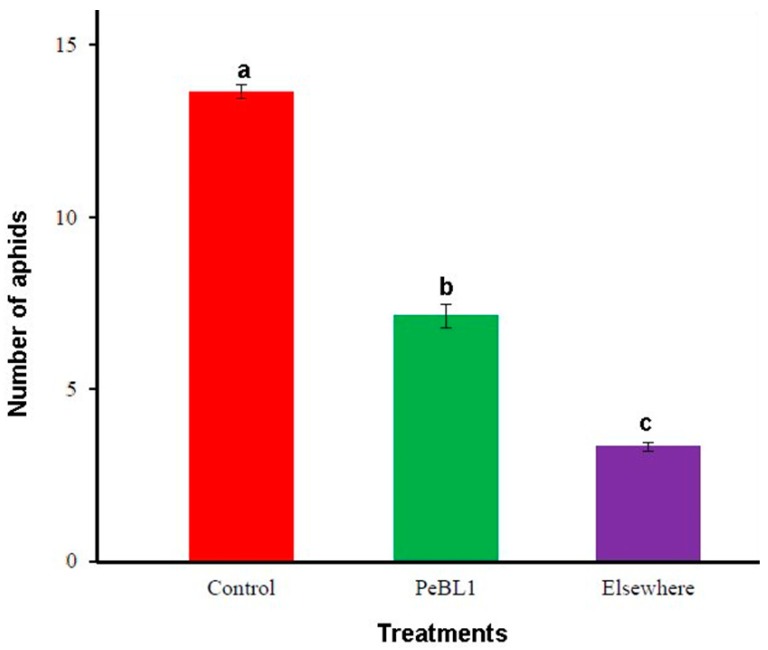
Colonization of *M. persicae* on PeBL1-treated and control tomato seedlings after infestation (mean ± SD); data compared by Latin square design (LSD), one-way ANOVA and Levene’s test with SPSS 18.0. Different lower style alphabets letters indicate significant differences among treatments (*P* = 0.05).

PeBL1 induced resistance to the tomato aphid *M. persicae* in two ways. Firstly, PeBL1-treated tomato seedlings showed a population decrease; Table 1 shows the percentage decrease in population in the PeBL1 treatment compared with the buffer and control treatments. *M. persicae* preferred feeding on control tomato plants in the host selection test. One day after aphid inoculation and two days after plant spraying, the number of *M. persicae* colonizing PeBL1-treated plants (7.13 ± 0.34) was significantly lower than the control (13.65 ± 0.18) and “Elsewhere” is colonization of aphid at places other than the buffer- and PeBL1-treated areas. Some aphids, based on their feeding behavior, showed colonization in areas opposite to that treated with buffer and PeBL1 (Figure 2). Secondly, in case of PeBL1 treatment, tomato aphid developmental time was more extended as compared to control, while daily reproductive abilities of *M. persicae* that fed on PeBL1-treated seedlings were decreased (second and third nymphal instars). Furthermore, second and third generations showed lower growth rates (Table 2).

### 3.3. Influence of PeBL1 on Nymphal Development Time

Factorial analysis showed an impact of different concentrations of PeBL1 $\left(F_{5,468}=77.84;p<0.0000\right)$ at three different temperature regimes$\;\left(F_{2,468}=158.43;p<0.001\right)$ and of their interaction$\;\left(F_{10,468}=1.61;p<0.0079\right)$ on the overall developmental time of *M. persicae* as shown in Table A2 of Appendix A. A differential trend was found for the effect of the protein elicitor on nymphal development time, at different temperature regimes. The developmental time of each nymphal instar was prolonged with increasing concentrations of PeBL1. Maximum nymphal developmental time was 3 days for 1st instar and 3.7 days for 4th instar nymphs for high concentration (88.72 µg mL^−1^) at low temperature (16 °C). Minimum nymphal development time 1.6 days was recorded for 1st instar for low elicitor concentration (22.17 µg ml^−1^) at high temperature regime (27 °C). In buffer-treated (control) plants, the nymphal development time varied from maximum 2 days for 4th instar, at 16 °C, to minimum 1.1 day for 1st instar, at 27 °C. In water-treated (control) plants, there was no significant increase in the nymphal development with 1.8 days for 4th instar at 16 °C, and 1 day for 1st instar, at 27 °C.

**Figure 3:**
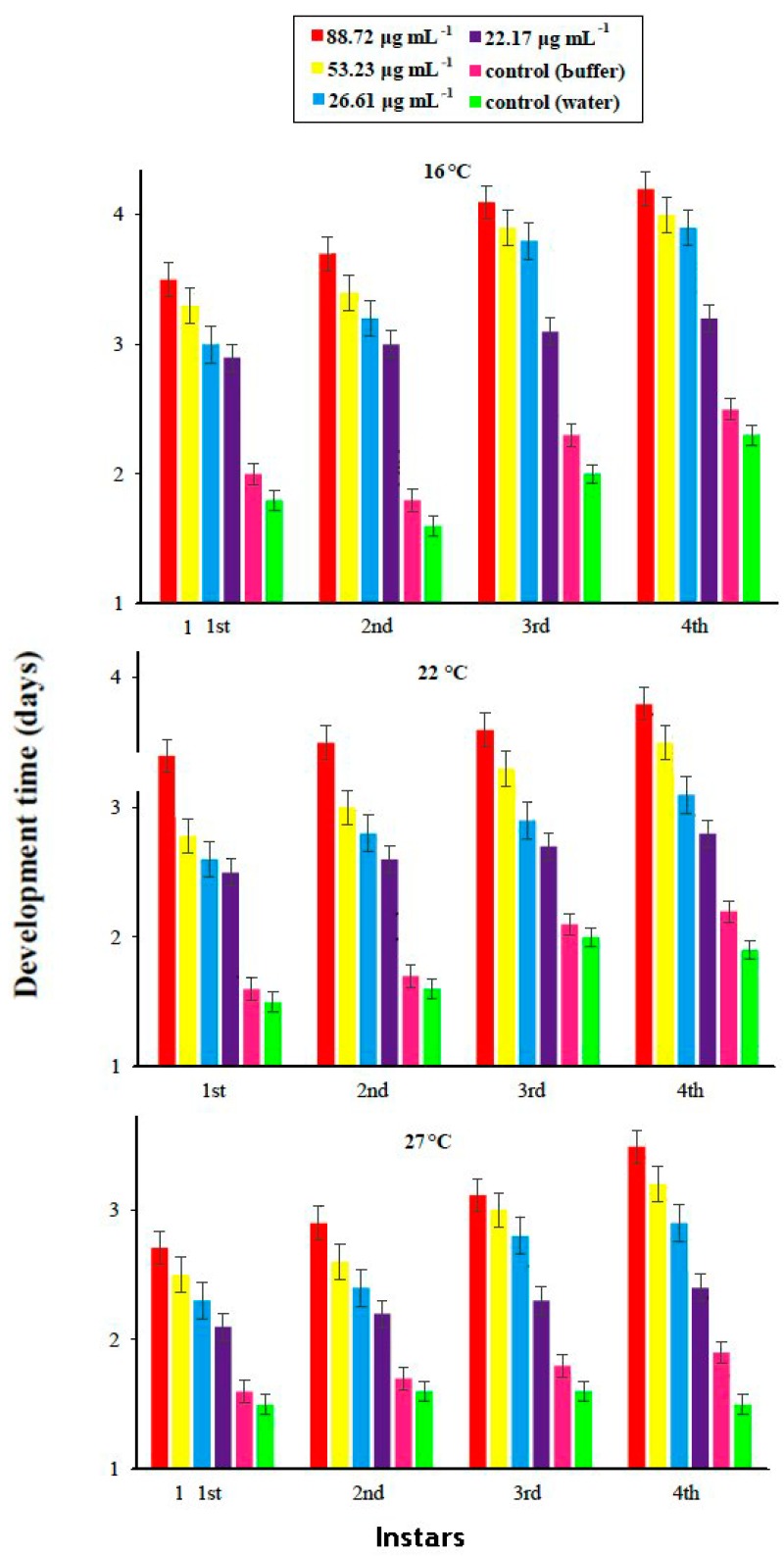
Mean development time (±SE) of different nymphal instars of *M. persicae* on tomato plants in response to the application of the PeBL1 elicitor protein, at different temperature regimes (*n* = 10). Different characters above bars indicate significant differences among treatments (one-way factorial ANOVA; LSD at α = 0.05).

In general, the nymphal development time of all instars at low temperature (16 °C) was higher at medium or high temperatures. Mostly, maximum elongation in time was observed for 4th instar at every temperature and concentration of elicitor. However, the influence of concentration of PeBL1 elicitor showed significance for the 1st $\left(F_{5,162}=77.50;p<0.0001\right)$, 2nd $\left(F_{5,162}=97.06;p<0.0000\right)$, 3rd$\;\left(F_{5,162}=80.21;p<0.002\right)$, and 4th instar $\left(F_{5,162}=74.27;p<0.001\right)$. Likewise, temperature regimes also had a significant impact on the nymphal development time of 1st $\left(F_{2,162}=52.27;p<0.001\right)$, 2nd $\left(F_{2,162}=38.60;p<0.001\right)$, 3rd $\left(F_{2,162}=43.29;p<0.001\right),$ and 4th instar$\;\left(F_{2,162}=40.97;p<0.001\right)$ aphids. After all, the nymphal development time did not show any fluctuation with their mutual interaction (Figure 3).

### 3.4. Effect of PeBL1 on Aphid Fecundity

**Figure 4:**
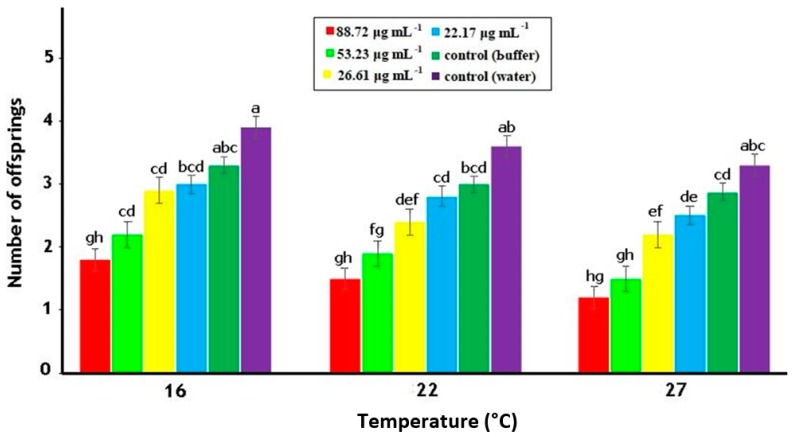
Average fecundity (mean ± SE) of *M. persicae* on tomato plants in relation to various PeBL1 elicitor concentrations, at different temperature regimes (*n* = 10). Letters on each bar show significant differences among treatments (one-way ANOVA; LSD at α = 0.05).

Data showed that the fecundity of *M. persicae* adults was significantly influenced by the concentrations of PeBL1 $\left(F_{5,162}=33.59;p<0.0001\right)$ and temperature regimes$\;\left(F_{2,162}=9.12;p<0.0006\right)$ as shown in Table A3 of Appendix A. Less offspring were produced by *M. persicae* individuals that fed on PeBL1-treated plants, as compared to those that fed on the positive (water) and negative controls. Moreover, the maximum fecundity was noted at minimum temperature (16 °C) while minimum fecundity was noted at maximum temperature (27 °C) (Figure 4).

### 3.5. Effect of PeBL1 on Tomato Leaves

The surface of tomato leaves was significantly modified by the PeBL1 protein. Seedlings treated with PeBL1 showed more trichomes than controls (PeBL1 treatment, 90.71 ± 1.20 mm^−2^; control, 48.49 ± 0.36 mm^−2^; *P= 0.05*). A more refined wax structure was formed that gave rise to a much more refined surface environment, which is a trait known for being unfavorable for the aphid colonization and other behaviors [28].

### 3.6. SA, JA, and ET Accumulation in Seedlings Treated with PeBL1

**Figure 5:**
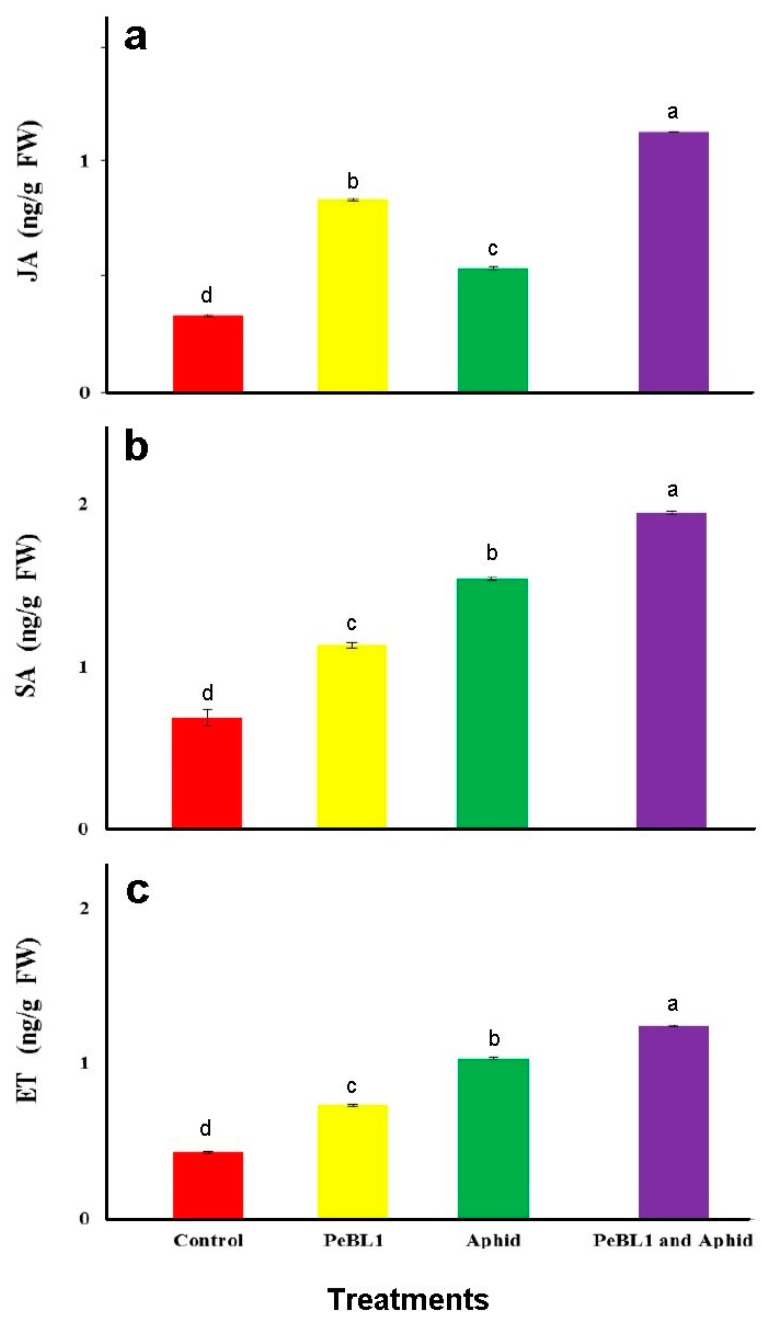
Contents in tomato seedlings (mean ± SD). Treatment with PeBL1 was carried out one-day after spraying. In both treatments, the aphids were inoculated one-day after seedlings were sprayed, and the samples were collected one-day after inoculation. (Data were compared by LSD, one-way ANOVA, and Levene’s test using SPSS 18.0. Lower letters show significant differences among various treatments performed in jasmonic acid (JA), salicyclic acid (SA), and ethylene (ET), *P* = 0.05.).

To analyze relation of JA with SA and ET with the cuticular wax deposition and increase in trichome density, PeBL1, the aphid infestation, or both were analyzed for JA, SA, and ET seedlings’ contents. PeBL1 showed very high accumulations of JA, SA, and ET in seedlings. The content of JA in seedlings treated with PeBL1 alone (0.83 ± 0.00 ngg^−1^ FW) or aphid infestation alone (0.54 ± 0.01 ngg^−1^ FW) was significantly higher than in controls (0.33 ± 0.01 ngg^−1^ FW). On the other hand, it was lower than that in seedlings treated with both PeBL1 and aphid feeding (1.13 ± 0.00 ngg^−1^ FW). The same was observed for SA accumulation (control, 0.69 ± 0.05 ngg^−1^ FW; PeBL1 treatment, 1.13 ± 0.02 ngg^−1^ FW; aphid infestation, 1.54 ± 0.01 ngg^−1^ FW; PeBL1 and aphid infestation, 1.95 ± 0.01 ngg^−1^ FW) (Figure 5).

All three signaling pathways were shown to participate in aphid-induced resistance in tomato [29]. Furthermore, JA, SA, and ET in PeBL1-treated seedlings accumulated, suggesting that the defense response in tomato plants was at least partially induced by the protein elicitor. JA, SA, and ET inductions are known to be affected by numbers, infestation time, and aphid species [30,31].

### 3.7. Relative Fold Change of Defense-Related Expression

**Figure 6:**
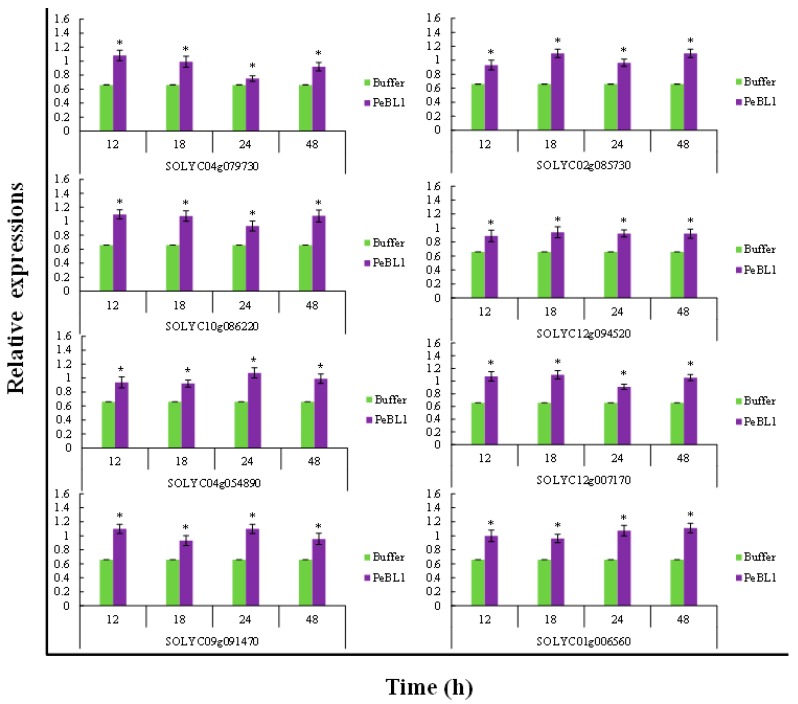
Relative expression of the JA pathway found at various time intervals after treatment with PeBL1 elicitor and aphid infestation. For each gene, an asterisk on bar shows a significant difference from buffer control, by Student’s *t*-test (*p* < 0.05).

**Figure 7:**
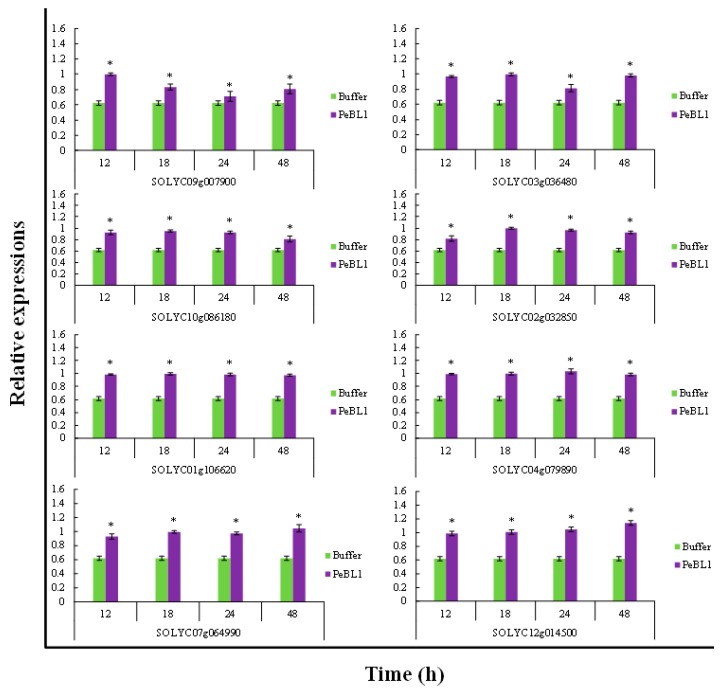
Relative expression of plant defense from SA pathway found at various time intervals after treatment with PeBL1 elicitor and aphid infestation. For each gene, an asterisk on bar shows a significant difference from buffer control, by Student’s *t*-test (*p* < 0.05).

**Figure 8:**
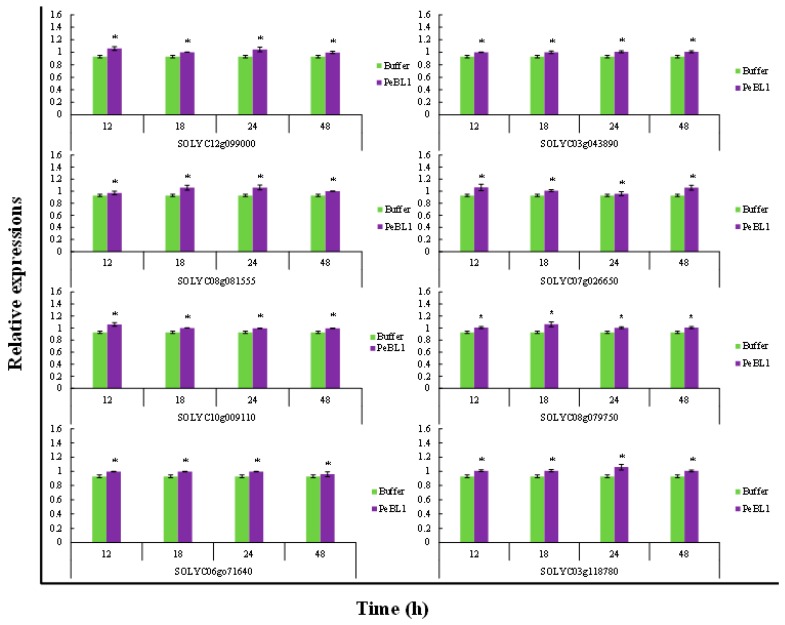
Relative expression of plant defense from ET pathway found at various time intervals after treatment with PeBL1 elicitor and aphid infestation. For each gene, an asterisk on bar shows a significant difference from buffer control, by Student’s *t*-test (*p* < 0.05).

**Figure 9:**
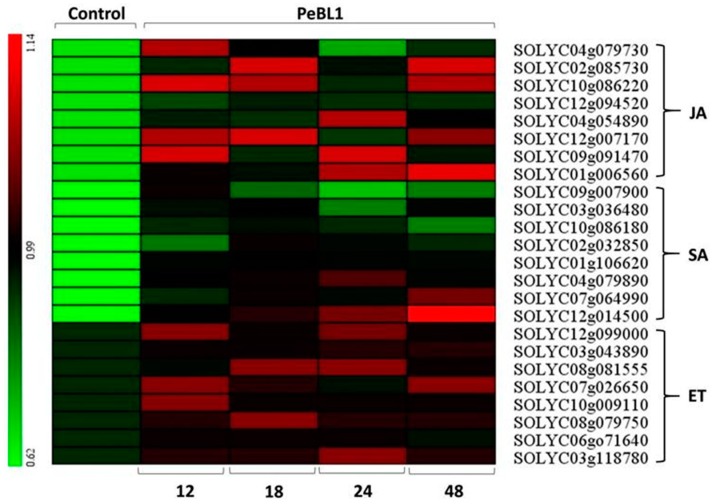
Heat map for expression of genes associated to the JA, SA, and ET showing the relative expression levels at 12, 18, 24, and 48 h after application of PeBL1 elicitor and aphid infestation. Scale color bar in picture shows high (red) to low (green) gene expression. Heat map obtained with the multiple experiment viewer software (MeV, version 4.6.0).

PeBL1 increased the defense response in tomato seedlings. All marker genes were up regulated by PeBL1 treatment, showing transcripts statistically more expressed than in the control. It was considered that induced resistance was caused by the aphid infestation and enhanced by PeBL1. Although genes involved in JA pathway were moderately expressed, all JA-associated genes were up regulated after 12, 18, 24, and 48 h of aphid infestation (Figure 6). Similar trends were observed for all SA- and ET-associated genes that were significantly up regulated and significantly different from control samples for all observation times (Figure 7 and Figure 8). Heat map of the expression levels of all 24 genes (Figure 9) suggest that resistance against aphid was due to increased transcription of the JA, SA, and ET genes.

## 4. Discussion

Use of elicitors represents a novel biological pest management technique, as they play a vital role in defense and signaling mechanisms of plants under attack of sap-feeding insects [32]. Various strains of *B. laterosporus* have shown different, broad-spectrum, antimicrobial activities against microbes such as bacteria and fungi, acting as antimicrobial peptides. They can enter the cell and be relocated in the cytoplasm and nucleus where they can interrupt the synthesis of proteins by intermingling to DNA and RNA [33]. Pathogenic bacteria and fungi, either necrotrophic or biotrophic, constitute an important source of elicitors such as PAMPs or MAMPs [34]. This study showed potential activity of PeBL1 derived from *B. laterosporus* A60 strain for the control of *M. persicae*. Previously, other studies showed that the application of chemical elicitors such as methyl jasmonate, benzothiadiazole, and other plant defense proteins such as proteinase inhibitors significantly reduced the activity of herbivore pests in tomato crop [35]. Data from this study validate previous findings showing that the soybean aphid *Aphis glycines* was reduced up to 40% with the use of methyl salicylate elicitor [34]. Bioassays data demonstrated that the population development was significantly slower on PeBL1-treated plants as compared to the buffer-treated control. Previous studies indicated a negative impact of exogenous applications of different elicitors, including methyl jasmonate (MJ), JA, and benzothiadiazole (BTH), on the population growth and fitness of different aphid species, an effect confirmed by the present findings [35]. Likewise, a biocontrol potential of this entomopathogenic bacterium has been found versus several Diptera, Coleoptera, and Lepidoptera, as well as versus mollusks and nematodes [23,24].

The present study revealed the potential of PeBL1 for the suppression of sap-feeding herbivores, affecting growth parameters and population performance. Trichomes are first lines of physical resistance against herbivores and pathogenic microorganisms. These hair-like appendages of plant epidermal cells [36] affect herbivore behavior, morphology, and density. *Solanum* spp. tests have shown a role of trichomes in defense, i.e., seven types of trichomes with two important effects in defense [37]. First, a plant surface constitutes a physical barrier because its dense hairs mat confers resistance, limiting the possibility of feeding and reducing the access of insects to the surface. Excessively hairy plants, such as *S. hirsutum*, are avoided by *M. persicae*. Trichomes are also associated with fundamental defense in tomato plant, as unicellular or multicellular hairs appendages arising from epidermal cell cover the surface conferring resistance to several pests due to the plant “pubescence.” Leaf beetle (Coleoptera: Chrysomelidae) colonization with dense trichomes was reduced in soybean pods as compared with the trichome-removed pods, which attracted more beetles [38].

PeBL1-treated seedlings and leaves possessed more trichomes as compared to controls. PeBL1-treated tomato seedlings and leaves with increased trichomes numbers were supposedly disadvantageous for aphid reproduction and colonization. Non-glandular, high-density, tomato trichomes negatively affected the feeding behavior of the Colorado potato beetle *Leptinotarsa decemlineata* [39,40]. The cell wall is another vital part of physical barrier lignin, underpins plant resistance, and is an indicator of the cell-wall enhancement [41,42]. Aphid tolerance in chrysanthemum was improved by an increased lignin content [43]. Physical defenses in plants include trichomes and wax production, in response to biotic or abiotic stresses. Their formation can be induced by direct damage, i.e., as induced by leaf-cut, methoxyfenozide, and manganese [44,45]. Cuticular wax deposition and trichome density may be also affected by application of exogenous phyto-hormones, MJ or JA, as shown in Arobidopsis and tomato, respectively [46]. Wax deposition in *B. napus* was induced with SA application [36]. Therefore, it can be speculated that accumulations of SA and JA in PeBL1-treated tomato seedlings were related to the increased density of trichomes and deposition of cuticular wax.

However, various proteinase inhibitors were produced by chemical elicitors previously described in tomato plants [47]. Furthermore, negative effects were exerted by the application of PeBL1 elicitor on the aphid fecundity. PeBL1-treated plants produced much lower number of aphids as compared to buffer-treated and control seedlings. Results are in line with previous studies evidencing that exogenous application of SA and MJ induced a lower mean lifetime fecundity in aphids [34,48]. Likewise, optimum temperature, i.e., 22 °C, showed maximum fecundity of aphids while higher temperatures, i.e., 27 °C showed minimum fecundity, reduced metabolic rate at high temperature being responsible for the effect [48,49]. Similarly, analysis of variance showed that nymphal developmental time was prolonged in plants treated with PeBL1 as compared to control. Also at lower temperature (16 °C), maximum nymphal development time was observed, confirming that an increase of one degree in temperature affects the insect’s life cycle to become shorter [50]. Further studies are required to understand the underlying mechanism induced by PeBL1 in tomato plants, in particular, concerning its effect on fecundity and nymphal development time.

Additionally, JA, SA, and ET increased transcription of marker genes, which suggest they play a role in aphid resistance in tomato. Aphid infestation in Arabidopsis significantly increased the transcripts of SA-related genes *(PR5, PR1*, and *BGL2)* and JA marker genes *(PDF1.2* and *LOX2, LOX12).* Actin is a structural component in the plant cell wall [51], which undergo depolymerization through regulation in cell and cross-linking [52]. Actin depolymerization negatively correlated with aphid fecundity and population [53]. JA, SA, and ET molecules confer a certain degree of resistance against insect herbivory and pathogenic attack, enhancing plants’ defense responses [35,54,55,56]. All key genes in this study associated with JA, SA, and ET showed significant and strong up regulation, in particular, the *AOS*, *AOC*, *OPR*, *4CL*, *ACX*, *AIM1*, *KAT2*, and *LOX12* marker genes for the JA pathway. Increased transcription of *AOS* (coding for allene oxide synthase) improved aphid resistance as shown in tomato by Thompson and Moran [54]. *AOC*, coding an allene oxide cyclase reduced the feeding activity and survival rate by increased crop resistance [57]. *PAL* and *4CL*, coding for a phenylalanine ammonia-lyase and a 4-coumarate–CoA ligase family protein, respectively, are involved in the construction of the cell wall, as shown in Arabidopsis [58]. *ACX* codes for an acyl-coenzyme A oxidase necessary for anti-insect defensive process and reproduction. *AIMI* and *LOX* code for fatty acid beta-oxidation multifunctional proteins. Lipoxygenase up regulation in JA occurs upon Pseudomonas inoculation in tomato plants [59]. *KAT2* codes A 3-ketoacyl-CoA thiolase, carrying out wound-activated responses, as shown in the biosynthesis of JA in wounded Arabidopsis plants [60]. *PAD4* codes for a phytoalexin-deficient 4-1 protein that mainly works in the balance of resistance (R) genes [61,62]. *NPR1* is a pathogenesis-related protein involved in systemic acquired resistance [63]. *SAMT* codes for S-adenosyl-L-methionine—a carboxyl methyl transferase that functions in systemic and local defense responses [64]. *SlERF3/LeERF3b* ethylene response factors are involved in stress response and significantly increase broad spectrum resistance in tomato [65]. *TAR 2* codes for tryptophan amino transferase in ethylene-mediated signaling functions in plant defense against bacteria [66]. Findings from this study confirm the activation by *M. persicae* of JA, SA, and ET pathways-associated genes [56,67].

## 5. Conclusions

Data showed aphid resistance in tomato with increased developmental time of each nymphal instar, associated to a lower fecundity of *M. persicae*. Aphid colonization was also affected by increased concentrations of PeBL1. The resistance factors were confirmed by increasing number of trichomes and quantity of wax, which mostly are involved in mechanical defenses. Physical defense response induced by PeBL1, JA, SA, and ET participated in a global plant physical response. However, some problems need to be resolved in future, such as “whether composition of wax influences or not the aphid behavior”; “how JA, SA, and ET function in induction of resistance”; and “whether or not other plant hormones are involved in this process.” However, the current study provided evidence that PeBL1 isolated from *B. laterosporus* strain A60 could be applied to tomato seeds and seedlings to protect plants from *M. persicae*.

## Figures and Tables

**Table 1:** Differences of *Myzus persicae* number in control, buffer-, and PeBL1-treated tomato seedlings.

Days After Aphid Inoculation	Control	Buffer	PeBL1
5	54.61 ± 0.04 b	62.92 ± 0.04 a	48.54 ± 0.40 c
10	109.22 ± 0.08 b	125.84 ± 0.08 a	92.43 ± 0.39 c
15	218.44 ± 0.15 b	251.86 ± 0.15 a	176.06 ± 2.09 c

Data compared by least significant difference (LSD), one-way analysis of variance, ANOVA, and Levene’s test with SPSS 18.0. Letters in rows show significant differences at different treatments; same day after aphid inoculation (*P* = 0.05).

**Table 2:** Time for development, mean reproductive ability, and growth rate of *M. persicae* in PeBL1-treated and control seedlings of tomato (mean ± SD).

Generations of *M. Persicae*	Td (Day)		No of Nymphs Per Day		r_m_	
	Control	PeBL1	Control	PeBL1	Control	PeBL1
1st	6.22 ± 0.29	6.1 ± 0.26	3.32 ± 0.08	3.16 ± 0.11	0.41 ± 0.02	0.39 ± 0.01 *
2nd	5.66 ± 0.17	5.95 ± 0.23	2.85 ± 0.14	2.64 ± 0.07	0.37 ± 0.01	0.33 ± 0.01 *
3rd	6.9 ± 0.36	6.53 ± 0.32	1.88 ± 0.15	1.5 ± 0.08	0.2 ± 0.006	0.17 ± 0.004

Td, development time; no of nymphs per day, average reproduction ability; r_m_, growth rate. (Data compared by LSD, one-way ANOVA, and Levene’s test with SPSS 18.0. Asterisks shows difference between PeBL1 and control *: *P* = 0.05.).

## Appendix A

**Table A1:** Pairs of primers of all test genes JA, SA, and ET involved in the plant defense.

Target Gene	Forward Sequence (5′ → 3′)	Reverse Sequence (5′ → 3′)
SOLYC04g079730	GCTACAATTCCCCTCGCATA	ACAGGTGGTGATGACGATGA
SOLYC02g085730	ATTTTCCTGCAAGAGCCAGA	TTCGTGCTTGATCAGAATGC
SOLYC10g086220	ACGAATTTCGAGTTGCTGCT	CTCATTTGCAACTGCTTCCA
SOLYC12g094520	GTTGATCCGGAGAGTGGAAA	ACTCTGCTGGAGGAACCTGA
SOLYC04g054890	GCAGCCTGCCTACAATTAGC	GCAAAATGGAATGCGTAGGT
SOLYC12g007170	CAACGGGCTACATCAAAGGT	TTGTTTGCTTTGTCCTGCTG
SOLYC09g091470	CTGATGGTGCTGGAGCTGTA	TTTGACAGCAGCTGGAATTG
SOLYC01g006560	AGAGGCGGTTCAACAAAAGA	CAATCGCCAAAGGTCTCAAT
SOLYC09g007900	TTTCGCTGAAGTGATGAACG	CTTAGTTGCTGCACGGATGA
SOLYC03g036480	GCCTTACATGGTGGCAACTT	GCCCTTGAAACCATAGTCCA
SOLYC10g086180	GGATCCTTTGCAGAAACCAA	GCCACCATGTAAAGCCTTGT
SOLYC02g032850	AACATTTGGCTCTCCAATGC	CTCCCAGAGGGACAAAATGA
SOLYC01g106620	TACTCAGGTGGTGTGGCGTA	TCAAAAGCCGGTTGATTTTC
SOLYC04g079890	AATTCACAATCGCGAAAACC	CCTTTGGACAGATCGCATTT
SOLYC07g064990	CGATCTACCGTCGAACGATT	CAACACTATCTCCGGCACCT
SOLYC12g014500	ACATCCCAGTGTATGCACCA	GTCCCCGAGTTGTTCACCTA
SOLYC12g099000	TTTGTCATTGGTGGACCTCA	CTTCTGGCAAGACCGTTAGC
SOLYC03g043890	TACTACCCGGGATTTGATCG	CGTTACGCGTTAATGTGGTG
SOLYC08g081555	AATGATGGTCATGGGGAAAA	ATTCACCACCCATTCTTGGA
SOLYC07g026650	CAACACAGACTGGGAAAGCA	GGCCAAGGTTCTCACACATT
SOLYC10g009110	CTTTCGATACTGCGGAGGAG	CGGACTCCGATTGATTTCAT
SOLYC08g079750	GGGTGGGGGTCATCTATTCT	AAGCCCCCACTACTTCTGGT
SOLYC06go71640	GGAAGCAAATGGGAGACAAA	GCATTCGATGGAGAGAGAGC
SOLYC03g118780	CTGGGCAATGAACAGGATTT	AAGCACTTTTGCAAGCCACT
18S	GTGACGGGTGACGGAGAATT	GACACTAATGCGCCCGGTAT

**Table A2:** Effect of temperature and PeBL1 elicitor protein on the nymphal development time of *M. persicae* (ANOVA).

SOV	Df	1st Instar			2nd Instar			3rd Instar			4th Instar			Df	Overall		
		MS	F-Value	*P*-Value	MS	F-Value	*P*-Value	MS	F-Value	*P*-Value	MS	F-Value	*P*-Value		MS	F-Value	*P*-Value
Conc.	5	10.78	77.50	0.0000	13.39	97.06	<0.000	16.12	80.21	0.0000	16.57	74.27	<0.0000	23	13.64	77.84	<0.0000
Temp.	2	5.32	52.27	<0.0000	5.32	38.60	<0.000	8.70	43.29	<0.0000	9.14	40.97	<0.0000	2	27.78	158.43	<0.0000
Conc. × Temp.	10	0.34	0.37	0.0095	0.36	2.65	0.0052	0.311	1.55	0.1259	0.13	0.60	0.81	46	0.281	1.61	<0.0079
Error	162	0.13			0.1380			0.2011			0.2232			648	0.175		
Total	179													719			
Grand Mean/CV		1.99/18.73			2.05/18.08			2.34/19.15			2.48/19.02				2.21/18.88		

**Table A3:** Effect of PeBL1 elicitor protein and temperature on fecundity of *M. persicae* (ANOVA).

SOV	DF	SS	MS	F-Value	*P*-Value
Conc.	5	91.183	18.2366	33.59	0.0000
Temp.	2	9.902	4.9508	9.12	0.0002
Conc. × Temp.	10	0.554	0.0554	0.10	0.9998
Error	162	87.948	0.5429		
Total	179	189.587			
Grand Mean/CV	2.5646/28.73				
